# Unmet need for family planning among Syrian migrant women living in Turkey and its determinants

**DOI:** 10.1186/s40834-024-00277-9

**Published:** 2024-06-20

**Authors:** Sema Cifci, Sibel Icke, Sevil Hakimi

**Affiliations:** 1 Faculty of Health Science, Department of Nursing, Mardin Artuklu University, Mardin, Turkey; 2 Faculty of Health Science, Department of Midwifery, Mardin Artuklu University, Mardin, Turkey; 3 Faculty of Health Sciences. Department of Midwifery, EGE University, Izmir, Turkey

**Keywords:** Immigrants, Refugees, Unmet need for family planning, Syria

## Abstract

**Introduction:**

Migrant women might be cannot benefit from health services sufficiently. The unmet need for family planning is among the pivotal indicators for measuring progress toward improving maternal and child health. The aim of this study was to identify the unmet need for family planning (UMNFP) among Syrian migrant women living in Mardin and its determinants.

**Material and methods:**

The study was conducted in Mardin. Data were gathered during home visits Data collection tools were socio-demographic and reproductive health questionnaires. The statistical analysis was performed using SPSS software. Qualitative variables were presented by number and percentage. Quantitative variables were presented by means (standard deviation). To determine, the determinants of UMNFP binary logistic regression was used.

**Results:**

The result of this study showed that prevalence of UMNFP was 35%. Woman’s low educational level (OR:5.42, CI95%:2.43–8.94), history of un intended pregnancy(OR:1.43, CI95%:1.1–1.94) and induced abortion (OR:1.76, CI95%: 1.41–2.21), not having husband’s regular job(OR: 2.24, CI95%:1.92–3.78) and lack of woman`s autonomy in decision related to use of contraception methods(OR:3.21, CI95%: 1.78–6.12) were determinants of UMNFP.

**Conclusion:**

The prevalence of UMNFP among Syrian immigrants living in Mardin was considerable. Understanding the challenges and the barriers impacting use of contraception including cultural norms as well, as social and language obstacles are essential to decrease UMNFP.

## Introduction

The provision of services that facilitate reproductive health goals, such as planned pregnancy, birth spacing, desired number of children, and informed selection of contraceptive methods, makes family planning a crucial aspect of public health [1]. Women’s reproductive health is significantly influenced by decisions related to family planning. The unmet need for family planning (UMNFP) refers to the percentage of women of reproductive age (whether married or in a consensual union) who are not using any form of contraception but desire to delay their next pregnancy or have no intention of having more children [2]. Additionally, this includes all postpartum amenorrheic women (whether married or in a consensual union) who are not utilizing family planning and whose most recent birth was unintended or unplanned [3]. Assessing the prevalence UMNFP among women in their reproductive years is crucial for monitoring advancements in maternal and child health [4].

Due to factors such as war, violence, climate change, or poverty, individuals may experience displacement either within their own country or across borders. As reported by the United Nations High Commisioner for Refugees (UNHCR), the number of displaced individuals exceeded 100 million by mid-2022. Notably, Turkey is home to the largest population of refugees, with approximately 3.7 million people seeking refuge within its borders [5].

Migrants and refugees often face significant barriers in accessing adequate healthcare services. These challenges arise from factors such as financial constraints, limited or no health insurance coverage, language barriers, and inadequate health policies specifically addressing the healthcare needs of migrants. These circumstances can contribute to the deterioration of their health condition and hinder their ability to receive the necessary medical care and support [6, 7]. Available evidence indicates that migrants are likely to encounter unmet reproductive health needs, which can include low rates of contraceptive utilization, insufficient knowledge about contraceptive methods, and a higher likelihood of unintended pregnancies. These factors highlight the importance of addressing reproductive health disparities and providing targeted support and education to migrant populations to ensure their reproductive health rights are met [8–10].

Syrian women exhibit high rates of pregnancy, but unfortunately, they often face challenges in accessing prenatal and postnatal care services. This results in low rates of receiving the necessary healthcare and support during both the antenatal and postnatal periods. Addressing these barriers and improving access to comprehensive maternal healthcare services is essential to promote the well-being of Syrian women and their infants [11]. Within the Syrian population under temporary protection in Turkey, approximately 24% fall within the reproductive age group. This signifies a significant portion of the population that requires specific attention and support in terms of reproductive health services, including family planning, maternal care, and reproductive health education. Adequate resources and targeted interventions are essential to address the unique reproductive health needs of this population effectively [12]. Limited research has been conducted on the UMNFP among Syrian refugees residing in Turkey. However, a study conducted by Col et al. revealed an estimated UMNFP rate of approximately 35% within this group. This finding highlights the importance of addressing the reproductive health needs of Syrian refugees in Turkey and implementing interventions to ensure access to comprehensive family planning services, education, and support. Further research is warranted to gain a more comprehensive understanding of UMNFP among Syrian refugees in Turkey and to inform targeted interventions [13].

The purpose of this study was to examine and understand the unmet need for family planning among Syrian migrant women residing in Mardin, Turkey, as well as the various factors influencing this need.

## Materials and methods

### Study design and population

This study is part of a broader research initiative that examines the utilization of contraception and the prevalence of domestic violence among married Syrian refugee women residing in the Mardin region. Mardin, situated in Southeastern Turkey, is recognized as a Kurdish region within the country. The study specifically focuses on married Syrian refugee women aged 15 to 49 living in Mardin. It is worth noting that, according to official records from the Turkish government, the population of Mardin city is approximately 850,000 individuals. By conducting this study in Mardin, researchers aim to gain insights into the contraceptive practices and experiences of domestic violence among Syrian refugee women in this particular region [14]. Furthermore, Syrian refugees make up nearly 10% of the total population of the city [15]. The study sample consisted of 380 Syrian women, which was determined using the EpiInfo program. The sample size was calculated based on an estimated prevalence of 50% eligible women and a 95% confidence interval (CI). Due to the unknown and estimated nature of the Syrian population, probability sampling was not utilized, and instead, snowball sampling was employed to reach the desired sample size. Ultimately, the study was completed with 401 participants, accounting for potential attrition.

To locate the participants, information obtained from the migration office of Mardin was used to determine their postal addresses. Home visits were then conducted by the first two authors (SC and SI), accompanied by an interpreter, to administer the questionnaires.

The data collection tools utilized in the study included a socio-demographic questionnaire and a reproductive health questionnaire. The comprehensive questionnaire covered various aspects, including sociodemographic information, reproductive history, knowledge about different contraception methods, and the sources of information. To ensure the validity of the questionnaire, content analysis was employed. The research team carefully reviewed the questionnaire items to assess their relevance, simplicity, and potential duplication.

### Statistical analysis

The statistical analysis of the data was conducted using SPSS software version 18. Qualitative variables were presented in terms of frequency and percentage, while quantitative variables were presented as means with standard deviation. To assess the determinants of UMNFP, binary logistic regression was employed. Initially, associations between socio-demographic and reproductive characteristics were examined using statistical tests such as chi-square, Fisher’s exact test, and Student’s t-test. Variables with a *p*-value of less than 0.2 were included in the regression model, and the final calculations were performed using the ENTER model.

## Results

**Fig. 1:**
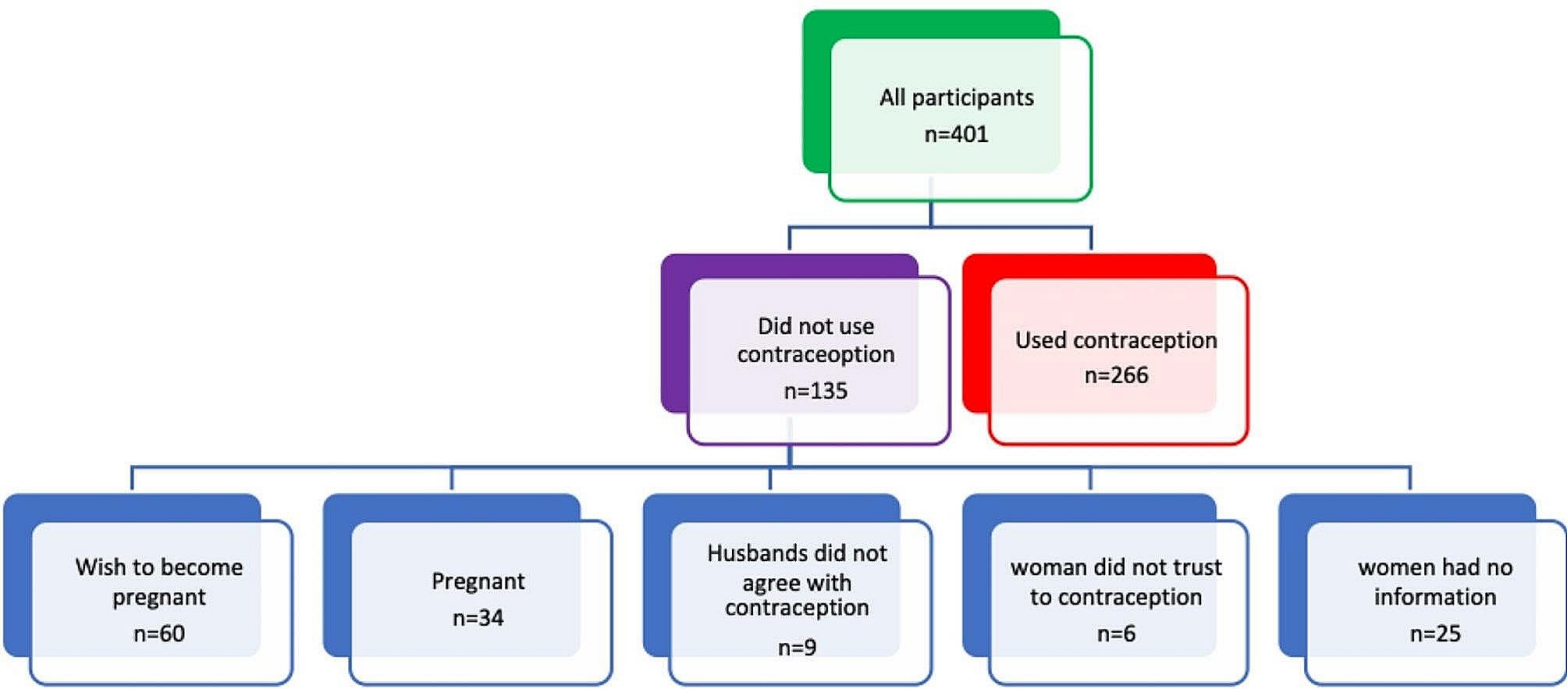
Participants allocation

The study was carried out from May to July 2022. Figure 1 illustrates the allocation of participants in the study. The mean age of the participants was 29.95 years, with a standard deviation of 8.25. Out of the 401 participants, 266 (66.33%) reported using contraception methods. Additionally, 40 women were pregnant at the time of the study.

Table 1 provides a summary of the demographic characteristics of the participants, while Table 2 presents an overview of their reproductive characteristics. These tables offer detailed information on various factors such as age, marital status, education level, number of children, and contraceptive use among the participants.

**Table 1:** Sociodemographic characteristics of participants

Characteristics	Number (n)	Percentage (%)
**Age group**		
15–18 years	12	3.0
19–34 years of age	280	69.8
35 and older	109	27.2
***Type of marriage***		
Civil marriage Religious marriage	350 51	87.3 12.7
***Education level***		
Illiterate < 9 years formal education 9-12years formal education > 12 years formal education	25 194 89 93	6.2 48.4 22.2 23.2
***Husband’s education level***		
Illiterate < 9 years formal education 9–12 years formal education > 12 years formal education	15 211 95 80	3.7 52.6 23.7 20.0
***Working in a regular job***	31	7.7
***Husband working in a regular job***	338	84.30
***Substance addiction***	9	2.2
***Husband’s substance addiction***	11	2.7
***Income level***		
Income lower than expenses Income equal to expenses Income higher than expenses	112 259 30	27.9 64.6 7.5

**Table 2:** Reproductive characteristics of participants

Characteristics	Number (n)	Percentage (%)
***Age at marriage, years*** ***Mean (SD)***	21.2	3.7
***Age of first pregnancy, years*** ***Mean (SD)***	21.0	6.6
***Having children***	369	92.0
***Number of children*** ***(*** *n* *** = 373)***		
1–2 child	215	57.6
≥ 3	158	42.4
***History of unplanned pregnancy***	154	38.4
***History of induced abortion***	33	8.2
***Interval between the last two pregnancies***		
Less than two years	98	34.1
***Reason for not using contraception (*** ***n*** *** = 135)***		
I want to have children	60	44.8
My husband does not want to use it	9	6.2
I do not trust its protectiveness	6	4.5
Have not information	25	18.6
Pregnancy	34	25.4
***Current use of any methods***	266	66.6
Reason for choosing that method* ( *n* *** = 266)***		
Reliability	97	36.5
Easy access	21	7.9
Easy application	63	23.6
Less side effects	85	32.0
Decision-maker about using contraception* ( *n* *** = 266)***		
Wife	18	6.8
Husband	7	2.6
Both together	232	87.2
Family elders	9	3.4
***talking about contraception with husband***	344	85.8

The study found that a significant proportion of the participants (80.5%) were aware of at least one contraceptive method. The most well-known methods were oral contraception pills (OCPs) (80.9%), intrauterine devices (IUDs) (65.8%), and condoms (62.3%). Among the participants, the most prevalent method used for contraception was the IUD (26.3%), followed by male condoms and the calendar method (each at 21%). Approximately 17% of the participants relied on the withdrawal method for contraception. Although there was considerable awareness about OCPs, only 13.5% of participants reported using them as a family planning method.

The study also examined the prevalence of unmet need for contraception among young married women, which was found to be 35.0% (95% confidence interval 33.8–37.1%).

Regarding sources of information on contraceptive methods, only a small proportion of participants (3%) reported obtaining information from books/magazines. Contributions from radio and television were limited (4.2%), while the internet played a moderate role (36.5%). The majority of participants (73.3%) received information from neighbors and relatives, while doctors and midwives/nurses were the source of information for 70.1% and 55.9% of women, respectively.

Table 3 presents the determinants of unmet need for family planning. Factors such as low educational level, history of unintended pregnancy and induced abortion, lack of regular employment for the husband, and limited autonomy of women in decision-making regarding contraceptive use were identified as determinants of unmet need for contraception.

**Table 3:** Determinants of unmet need for family planning

Variable	OR^±^	CI 95%^¥^	P^∞^
Educational level < 9 years formal education > 9 years formal education (refernec)	5.42 -	2.43 to 8.94 -	< 0.001
History of un intended pregnancy	1.43	1.1 to 1.94	0.023
History of induced abortion	1.76	1.41to2.21	0.012
Lack of Husband´s regular job	2.24	1.92 to3.78	0.032
Who is decided about use of contraception Women (reference) Anyone else of women	- 3.21	- 1.78 to 6.12	0.032
Hosmer and Lemshaw test	0.026		
Adjusted R Square	0.33		

^±^Odd ratio

^¥^Confeidence interval 95%

^∞^Binary logistic regression

## Discussion

This study focused on 401 Syrian women residing in Mardin, none of whom were living in refugee camps. Among these participants, approximately two-thirds reported using contraception methods. Among the 134 women who did not use contraception, around 45% expressed a desire to become pregnant, while 25% were already pregnant at the time of sampling. Consequently, the overall prevalence of unmet need for family planning in the study was approximately 35%.

Despite the fact that the Syrian women in this study lived in an urban area and had access to various contraceptive methods similar to Turkish women, the prevalence of unmet need for family planning was significant. The findings highlight the importance of addressing barriers to accessing reproductive health services and improving the availability and affordability of contraception options for Syrian women in Mardin.

The concept of unmet need for family planning serves as a valuable indicator for monitoring and evaluating family planning programs. It also plays a crucial role in assessing progress towards achieving universal access to reproductive health services. By identifying and addressing the unmet need for family planning, policymakers and healthcare providers can improve reproductive health outcomes and empower individuals to make informed decisions about their reproductive choices [16]. Indeed, UMNFP reflects a discrepancy between women’s reproductive intentions and their actual contraceptive behaviors [3]. The finding that the unmet need for family planning among Syrian women in Mardin is lower than that among migrant women (Iranian, Iraqi, Syrian, and Afghan nationalities) living in Germany suggests that there may be differences in access to and utilization of family planning services between these populations in different contexts [17]. Various factors could contribute to this difference. It could be related to variations in healthcare systems, availability and affordability of contraception, cultural norms and attitudes towards family planning, and the level of awareness and knowledge about contraceptive methods among these populations. However is higher than Myamnmar migrant women was living in Thailand [18]. Total unmet need estimated in our study is higher than Ozdemir et al., in rural and urban residents in Turkey. In line with our study, Col et al., in their systematic review showed that, prevalence of unmet need for family planning among Syrian refugees women living in Turkey is 35% [13]. The emergence of unmet need for family planning is influenced by a range of individual and community-level factors including such as women’s age, education, husband’s education, socioeconomic status, media, age at first marriage, number of living children, parity, household size, decision-making about health services, and knowledge about health services. These factors can vary across different populations and settings, but the literature has identified several common determinants associated with unmet need for family planning [19–21]. Our study identified several determinants of unmet need for family planning among Syrian women, including women’s education, history of unintended pregnancy, history of induced abortion, lack of autonomy in contraception decision-making, and the husband’s lack of regular employment. These findings are consistent with existing research conducted in various countries, which also highlight higher education as a protective factor against unmet need for family planning. Riaz’s and Asif’s work further supports these results. Higher education equips women with knowledge, decision-making abilities, and greater access to resources related to family planning, thereby reducing the likelihood of unmet need [22, 23].

Likewise, women’s autonomy in decision-making regarding contraceptive use is a significant factor that empowers women. Our study revealed that the absence of autonomy among women in decision-making increased the likelihood of unmet need for family planning by approximately three times. Additionally, our findings indicated that the lack of a regular job for husbands increased the odds of unmet need for family planning, exacerbating poverty. Asif suggests that wealthier families may have better access to modern contraceptive methods compared to their poorer counterparts [23]. In the current study, we found that a history of unintended pregnancy and induced abortion was associated with an increased likelihood of experiencing unmet need for family planning. These findings indicate that unmet need for family planning is influenced by individuals’ reproductive behaviors in the past. Therefore, it highlights the importance of addressing unmet need for family planning as it relates to individuals’ reproductive history. By recognizing and addressing the underlying factors associated with unmet need, such as past unintended pregnancies and induced abortions, we can work towards reducing this issue and promoting better reproductive health outcomes. Among participants used contraception, one-third used withdrawal and calendar methods despite their inefficacy. Higher use of traditional methods, higher un planned pregnancies, if no further effective family planning policies are taken [24].

In our study population, IUD emerged as the most preferred method among the modern contraceptive methods. This finding is consistent with the systematic review conducted by Col et al., which examined the contraceptive practices of Syrian migrants residing in Turkey. Their review also identified the IUD as the most prevalent modern contraception method utilized by this population. These findings suggest a preference for the IUD among Syrian migrants in Turkey, highlighting its importance and potential suitability in meeting their contraceptive needs [13]. According to the study conducted by Sato et al. on urban women living in Istanbul, the most commonly used method of contraception was withdrawal, followed by the use of IUDs. This finding suggests that withdrawal is the preferred contraceptive method among the study population, with IUDs being the second most utilized method. It is important to note that the choice of contraceptive method can vary among different populations and can be influenced by various cultural, socioeconomic, and individual factors [25]. Interestingly, none of the participants in the study considered vasectomy as a method of contraception. As a result, the prevalence of vasectomy in this particular study was found to be zero. This finding highlights a lack of awareness or acceptance of vasectomy as a contraceptive option among the study population. It is important to explore the reasons behind this observation, such as cultural beliefs, knowledge gaps, or personal preferences, in order to better understand and address the barriers to vasectomy utilization in this context. About 35% of participants who had at least two live birth, were spaced less than 24 months between the two last pregnancies which are less than WHO recommended inter- pregnancies interval [26]. Since we did not inquire about the intentionality of participants’ second pregnancies, it is not possible to calculate the unmet need for spacing between pregnancies in our study.

## Limitation

Our study has several limitations that should be taken into consideration. Firstly, the use of a purposeful sampling method may introduce potential selection bias. Secondly, the sensitivity of the study topic may have influenced the participants’ responses, leading to potential bias in questionnaire completion. To mitigate this issue, we employed native interpreters to assist participants in filling out the questionnaires. However, due to the subjective nature of the research, it remains uncertain whether the answers received were entirely truthful. Despite these limitations, this study provides a significant contribution to the global literature on family planning among asylum seekers and refugees, given the sizable sample size.

## Conclusion

In spite of the efforts made by the Turkish government and NGOs to provide free family planning services to immigrants [13], the prevalence of UMNFP among Syrian immigrants is significant. It is crucial to gain a better understanding of the challenges and barriers that affect the use of contraception among this population, including cultural norms, language barriers, and attitudes towards family planning. By addressing these factors, we can work towards reducing the occurrence of unwanted pregnancies and improving access to and utilization of family planning services among Syrian immigrants.

## Data Availability

No datasets were generated or analysed during the current study.
